# Vitalism as Pathos

**DOI:** 10.1007/s12304-016-9254-7

**Published:** 2016-02-25

**Authors:** Thomas Osborne

**Affiliations:** SPAIS (School of Sociology, Politics and International Studies), University of Bristol, 11 Priory Road, Bristol, UK

**Keywords:** Vitalism, Mechanism, Canguilhem, von Uexküll - Nietzsche

## Abstract

This paper addresses the remarkable longevity (in spite of numerous ‘refutations’) of the idea of vitalism in the biological sciences and beyond. If there is to be a renewed vitalism today, however, we need to ask – *on what kind of original conception of life should it be based*? This paper argues that recent invocations of a generalized, processual variety of vitalism in the social sciences and humanities above all, however exciting in their scope, miss much of the basic originality – and interest – of the vitalist perspective itself. The paper argues that any renewed spirit of vitalism in the contemporary era would have to base itself on the normativity of the living organism rather than on any generalized conceptions of process or becoming. In the terms of the paper, such a vitalism would have to be concrete and ‘disciplinary’ rather than processual or generalized. Such a vitalism would also need to accommodate, crucially, the *pathic* aspects of life – pathology, sickness, error; in short everything that makes us, as living beings, potentially weak, without power, at a loss. Sources for such a pathic vitalism might be found above all in the work of Georges Canguilhem – and Friedrich Nietzsche – rather than primarily in Bergson, Whitehead or Deleuze.

‘- [T]he will to power not a being, not a becoming, but a *pathos*, is the most elemental fact, and becoming, effecting, is only a result of this…’^1^

Vitalism is a remarkably persistent if obviously uneven tradition of thought in the biological, medical and social sciences as well as in the humanities and philosophy. Over recent decades, notably through the rediscovery and re-appropriation of Bergson and Whitehead, and via the more direct influence of Gilles Deleuze the possibilities for a vitalism of generalized becoming and process across both living organisms and material ‘nature’ have been elaborated and defended especially in the social and cultural sciences (*inter alia* Stengers 2011; Fraser et al. 2005; Greco 2005; Lash 2006; Deleuze 1991). This kind of processual vitalism aims to integrate various otherwise extraneous modes of thought from the history of biology to contemporary philosophy, cultural theory, the arts, information science and complexity theory. Its perspective is not infra-disciplinary (for instance privileging the biological sciences) but takes as its object the total processuality of becoming itself, unrestricted by the closure of limitation or fixed form – and this knows no limits, embracing all sorts of apparently ‘non-living’ domains, including those more traditionally associated with principles of mechanism rather than the *élan vital*. Typically this perspective is also what might be labelled an affirmative or ‘celebratory’ vitalism. It is, so to speak, semiotically optimistic, staging the values of process and becoming against the apparently reductive principles of erstwhile mechanism, mere ‘Being’, the specific organism, closure, disciplinarity and stasis.

On the other hand, the principle limit of such generalized kinds of vitalism seems to lie exactly in its lack of limits. Precisely in stressing the ubiquity of processual becoming there is a tendency to collapse everything into itself, into generalized *process*, thus perhaps losing anything much to do with what is in fact, so to speak, *originally* ‘original’ to life. However, rather than seek to offer up an elaborate critique of such processual kinds of vitalism, this paper seeks simply to present another, more limited, more deflationary kind of view. This is the understanding of the originality of life as *pathos*. Whilst this has complex and contestable Nietzschean roots in the idea of life as the will to power, most of our focus in what follows will be on the work of that ‘non card-carrying Nietzschean’ – and professed vitalist – Georges Canguilhem, although we shall return to Nietzsche himself, necessarily, in our final remarks.

## About the ‘Vitality’ of Vitalism

Canguilhem has been somewhat neglected by the proponents of generalised, processual vitalism, principally because Canguilhem was *not* a generalizing vitalist but, rather like Jakob von Uexküll in Riin Magnus’s excellent account of a particular strand in twentieth century vitalism, something of a concrete or ‘disciplinary’ one (Magnus 2008; le Blanc 2002).^2^ Trained in both philosophy and medicine, Canguilhem (1904–1995) was one of the foremost French intellectuals of the post-war years, but on a very different plane from the superstar roll-call of existentialist, structuralist then post-structuralist philosophers. His work was firmly rooted in the history of biological and medical ideas and in the implications of these for modern conceptions of science and medicine, as instanced in his first and possibly most important work, the *Essai sur quelques problèmes concernant le normal et le pathologique* (1943; Canguilhem 1989). Throughout his career, Canguilhem insisted on the irreducibility of the life sciences to the physical and other sciences, and tracing the career of vitalist conceptions and the links between life itself and the knowledge of life in the history of European thought perhaps most importantly in his *La Connaissance de la vie* (Canguilhem 2008a, originally published in 1952).

Instead of seeing vital principles everywhere, Canguilhem began with biological (and medical) facts and remained within what Magnus labels, in the context of an earlier strain of vitalist thought, a disciplinary organicism (Magnus 2008). Our argument will be that such disciplinary reductionism actually gave his vitalism more not less purchase than the generalizing, processual sort, which in turn perhaps goes some way to explaining the neglect of Canguilhem by process-inclined vitalists. For Canguilhem, though a vitalist himself, vitalism as a general, positive *doctrine* is strictly-speaking erroneous. There is no generalized life force as such (nor is mechanism straightforwardly ‘false’). Bergson or at least latter-day so-called ‘Bergsonists’, in this sense at least, were wrong. But, for Canguilhem, the doctrinal fallibility of vitalism as a ‘positive’ form of thought is in fact part of its essence – a guide to research that evades the reductionisms of doctrinal mechanism. In this sense we could even call Canguilhem’s a ‘negative’ or perhaps a sceptical vitalism. As Monica Greco has shown in her reflections on Canguilhem’s vitalism, it is integral to vitalism’s own ‘vitality’ that it is constantly refuted and made apparently obsolete, and Michel Foucault described Canguilhem’s vitalism not as a doctrine but as an *indicator* of problems to be ‘solved’ and ‘reductions to be avoided’ (Foucault 1989: 18). For Greco, vitalism is ‘a significant motor force… the trope through which, and in answer to which the life sciences have come to constitute their own domain distinct from those of physics and chemistry’ (Greco 2005: 17).^3^ If epistemologically-speaking, vitalism is false, or is at least constantly falsified, no doubt this is more or less straightforwardly because there is in fact no generalizable ‘essence’ to the question of life to which a dogmatic vitalism could ever be an answer. No one will ever discover *the* unique property that makes up life, simply because there is no such property. Although one can write a ‘history’ of it and one can characterize or describe it, life is not the kind of phenomenon that one can reduce to an equation or a formula even though it is possible to describe, broadly speaking, what is characteristic *of* it.^4^ But determination and descriptive characterization are different things. *Determination* of the concept of life, as opposed to its characterization, is not – to refer to a common parallel – like that of the determination of the constituents of water, and life certainly cannot be reduced to a chemical format such as two parts hydrogen, one part oxygen.

Perhaps vitalism even thrives on a certain level of un-knowing; that we know *of* life and its originality but not *what* life as such is. For instance, one would think that today the very existence of a discipline called exobiology necessitates that we have some idea, some characterization, however basic, of what we are looking for when we look for life on other planets. Yet such definitions tend to be somewhat circular or at least situated on our own, terrestrial, more or less actualized experience of life. The NASA Exobiology Programme stated in 1992 that: ‘Life is a self-sustained chemical system capable of undergoing Darwinian evolution’ – which is in fact a perspectival definition based on the particular experience of Earthly life (Raulin 2010). The search for the origins of life – in thermal vents, on asteroids, in chance mutation from one single organism – implies some basic characterization of what life is, and yet the very search – and research into ‘non-living’ forms of life such as viruses is parallel to this – often seems to un-do the very co-ordinates with which we began. It is almost as if the less we can be doctrinal and deterministic about our definitions of life, the more vitalism is necessary.

For Canguilhem, vitalism was indeed an ‘exigence’, a duty, a requirement rather than a specific intellectual doctrine with a determinate, generalisable content (Canguilhem 2008a: 62; Canguilhem 1965: 86).^5^ No doubt it constituted such a requirement for him precisely as recognition of the disciplinary integrity of the biological sciences. Philosophical vitalism is one thing, biological vitalism another. Although influenced – especially in his earlier work – by Bergson, there is little in Canguilhem’s historical studies that is redolent of generalizing, processual bio-philosophy. After mentioning the varied and perhaps improbable uses to which such generalizing bio-philosophy might be put, Canguilhem provides in ‘Aspects of vitalism’ (dating originally from 1947) a list of prominent vitalists taken from the scientific rather than the generalizing philosophical tradition; Hans Driesch and Constantin von Monakow for instance (Canguilhem 2008a: 61).^6^ For Canguilhem, Driesch’s ‘diurnal’ experimental vitalism (the kind of vitalism that he practically used in his work) was one (admirable) thing, his ‘nocturnal’, *philosophical* vitalism (which Canguilhem firmly rejects) quite another (ibid: 68–69). In science, metaphysical kinds of vitalism have no place beyond being on occasion a useful critical ideology to wield against the dogmatic reductions of some kinds of mechanism. But one can also argue, as Canguilhem in effect does, for vitalism on biosemiotic as opposed to bio-philosophical grounds (and indeed von Uexküll, who did not of course actually deploy the term biosemiotics any more than did Canguilhem himself, was a major influence here). However it is defined substantively, life consists of an active relation of an organism attempting to construct a workable milieu cut out from a potentially antagonistic environment and such a milieu is conducted not least from the signalling of meaning (cf. Kawade 2009: 206). Life, however we conceive it, is obviously strictly provisional so far as the organism is concerned; it is a balancing act between organism and environment. Of course, vitalism has been carried by different doctrines but whilst the *doctrines* fall away, the ethos of vitalism – the commitment to the idea of the specificity and irreducibility of life – itself remains, and indeed a mistaken ‘scientific ideology’ can be of inadvertent service to scientific activities (Canguilhem 1988). But this can really only be a historical argument after the fact; here, the vitality of vitalism is not something that can be dogmatically predicted but only *shown*.

So vitalism, for Canguilhem, was less a generalizable, ‘positive’ idea than a critical response to any form of thought that would reduce life to something non-vital and especially all mechanistic forms of reduction: ‘The rebirths of mechanism translate, perhaps in discontinuous fashion, life’s permanent distrust of the mechanization of life’ (Canguilhem 2008a: 73). It is this critical aspect that gives vitalism its resurgent character, its own vitality, so to speak. Vitalism itself has vitality for the simple reason that life, as Canguilhem says, is original which is to say not just unique but *originating* (2008a: 61; Greco 2005). This is where Canguilhem’s ‘disciplinary’ rather than generalising, processual perspective has most force; life is original, biology studies life, medicine ‘corrects’ life, adjusts it, renews it. But from this disciplinary foundation, much else follows in terms that are not strictly biological. Life anticipates problems of knowledge; to be alive is to pose problems in relation to one’s environment, presupposing the demand to appropriate that environment through all sorts of knowledges and technique. We have knowledge and technology because we have life. The basic, biological originality of life is the precondition of all that we know or can think; it governs our perspectives, it produces value and valuations. Life is perspectival, but necessarily and foundationally so. Life does not subscribe to some pre-vital concept of the normal but conditions the very idea of the normal; it is, in Canguilhem’s terms, *normative* (Canguilhem 1989). Life ‘invests’ in norms, creating norms, but it does not merely ‘conform’ to them as eternal or over-riding scientific ‘laws’. In this respect, life *invents* norms. Hence if not as substantive doctrine, vitalism persists as something like the critical background property of the very possibility of knowledge, technique, experience and normativity.^7^

## Mechanisms, Machines and Emergence

How far are we to take this, however? Sometimes Canguilhem’s perspective can seem to border on a biological *reductionism* rather than a biological foundationalism. They are different things. Canguilhem at least in his earlier post-war work appears to have insisted – largely no doubt for reasons to do with a concern for disciplinary autonomy, and so privilege, of biology – that his sort of vitalism had to be not, as he put it, merely regional or ‘divisional’ but reductive or more less imperialistic; ‘the term *vitalism* is appropriate for any biology careful to maintain its independence from the annexationist ambitions of the science of matter’ (2008a: 60; cf. Greco 2005: 18–19). Everything we can know must ultimately be reducible to the originality of life since it was life that sought to reduce the mutual inarticulacy of organisms and the environment through knowledge and technique. Canguilhem wanted to avoid any sort of vitalism that made life a break in the normal run of physical laws; that is, which ‘accepts the insertion of the living organism into a physical milieu to whose laws it constitutes an exception’ (Canguilhem 2008a: 70). He voiced reservations even about the ‘classical’ vitalism of Bichat or Bernard, who held in different ways that life is a sort of complex folding or interruption in nature, subject to physical forces and properties but with its own, as it were local, rules, amounting to a kind of regional vital ontology.

For Canguilhem, however, at least in his earlier work, this sort of vitalism was not enough. The fact that life is original meant for him also that it is fundamental, that the sciences of life condition all others; in effect, that biology, to put it a little bluntly, would be queen of the sciences. Today, let us admit that this kind of rather fundamentalist perspective seems somewhat overstated, or at least un-necessary.^8^ One can still reductively assert the disciplinary foundations of vitalism without being a biological imperialist as such. Perhaps there was a kind of excluded middle to Canguilhem’s approach here in his earlier work; to the perceived imperialism of the physical sciences he wanted to posit an alternative imperialism on the part of the biological sciences. Later on he modified this view quite significantly. By the time of the ‘New reflections’ (1963–1966) to *The normal and the pathological* (originally 1944), specifically, in the essay dealing, in part, with the information sciences, Canguilhem appears to be more accommodating to the idea, associated with Bichat and others, that life is a sequestration or folding from physical laws, invoking ‘a philosophy of life understood as opposition to inertia and indifference… [according to which] life gambles against growing entropy’ (Canguilhem 1989: 236). Meanwhile, in ‘Le concept et la vie’, also from 1966, there is reference to the concept of information albeit not to the information sciences as such (Canguilhem 1968a: 362–3).

Such references would seem to support the idea of continuity between the life sciences and the physical sciences, without for all that denigrating the *originality* of life as such. Today, in any case, the opposition between life and mechanism has been transformed. Often this opposition was subsidiary to – and dependent upon – that pertaining between idealism (or even something like spiritualism) and materialism. Nowadays few vitalisms would aspire to be overtly of a spiritualist sort, and even if Bergson is invoked it is the Bergson of duration and seldom that specifically of the *élan vital*, a term – though largely in fact corresponding to that of duration itself – has come to take on a distinct character in post-Bergsonian commentary as a kind of independent, transcendent force (cf. Fraser et al. 2005: 3). Certainly, any worthwhile vitalism today would have no need to be anything other than a resolutely materialist vitalism; ‘nothing happens in this world, not the flutter of an eyelid, not the flicker of a thought, without some redistribution of microphysical states’ (Quine 1981: 98). Similarly with mechanism; nowadays more than ever, vitalism can surely incorporate mechanism. If this was not Canguilhem’s own earlier view, perhaps it is the case that he himself then relied on a rather polemical, understanding of mechanism, to which he was thereby – rather unsurprisingly – opposed. In any case, we might nowadays stubbornly insist that *mechanism is not what it was*.

But in fact mechanism was scarcely what it was even when Canguilhem was writing his post-war reflections upon it. In the late 1920s and 1930s writers such as J.S. Haldane began to think of life as reducible neither to mechanism nor vitalism, but to be something of both – a specific form of *organization* (Haldane 1936; Magnus 2008; Bechtel 2007)..^9^ Haldane’s position was to accept the originality and irreducibility of the vital order but not to countenance doctrinal vitalism per se, still less the ‘spiritualist’ idea of a vital force. In this sense the crude vitalist-mechanistic oppositions that had animated the work of previous generations were already superseded by the time Canguilhem wrote some of his most important essays from the 1940s. And by now they are superseded from both sides of the debate, as if mechanisms had become vital and life mechanistic. The physical sciences have themselves become – as the processual vitalists have indeed recognised (Fraser et al. 2005) – more, so to speak, *oriented* towards life as a fundamental rather than incidental aspect of their concerns. Mechanisms are not so much hard and fast laws of the physical universe, but rather mechanisms in the sense of processes or tendencies; not so much cogs and wheels but tendencies that are dependent on their scale of interaction, subject to counter-effects and counter-tendencies, and as likely to be non-linear as linear.

In this context we might add that the discovery of the nano-scale has also led to a kind of two-way interpenetration of the physical and life sciences (Black 2014). The metaphor of life as a machine has moved radically away from the Cartesian model of the animal-machine evocatively described by Canguilhem in his (originally, 1947) essay ‘Machine and organism’ (Canguilhem 2008a: 75–97). It is not at all that the analogy has disappeared, but more as if nowadays machines tend to be conceived as being more like animals than animals like machines; and, more specifically, the science of soft machines and soft materials, with its language of membranes and surfaces rather than cogs and wheels resonates more with phenomena of life than Cartesianism or classical mechanics ever did (see Jones 2007). Today’s physical – mechanical – engineers of porous surfaces, nano-scales and soft membranes tend to regard life as a *model* rather than as a randomly formed, and thereby imperfect, *exception* to mechanistic laws of configuration, and this could be said to give new relevance to Canguilhem’s prioritisation of life over machine. This is not to say that nano-engineering must itself always be understood as an extension of a vitalist viewpoint, only that it is looking to the basic *originality* of life for some of its models. God is indeed a machinist in so far as s/he is a nanotechnologist. In organisms, claims Jones:

[t]he main structural elements are floppy, carbon-based polymers and even more transient assemblies of detergent-like molecules – little more than water-filled soap bubbles, in fact… [N]ature has done the best she can in the face of the difficult constraints imposed by evolution… [N]ature’s designs are in fact highly optimised’ (86–87).

But optimisation here should not mean finite or finalized, but – as in the emphasis well known from biosemiotics – flexible. Nature optimises in so far as nature improvises. When organisms are understood as machines they are still organisms *because* not *in spite* of the fact that they are possible to comprehend as machines, just as it appears that machines are increasingly mirroring – or at least being modelled upon – life; and as Canguilhem himself wrote, life itself is if not machine-like then *machinic* in so far as humans themselves are machinists (Canguilhem 2008a: 111); which is to say that they are makers or rather originators of machines.

Here Canguilhem’s thought is of course in some conjunction with the biosemiotic emphasis on machines as being extensions of the originating purposiveness of life (Kawade 2009: 211). Moreover, the existence of the nano dimension and also, in a different way, the micro world of quantum mechanics shows that there can be regionalizations of more overt or general physical laws that are more like foldings within such laws or in the case of the quantum universe substrata of them rather than entirely insulated ‘transgressions’ of them. No one would say that quantum mechanics has no status because of the continued existence of phenomena associated with gravity any more than one would deny on the basis of quantum behaviour the possibility of travelling to the moon. Cosmic existence it seems is subject to regional, ‘disciplinary’ reductionisms without, for all that, disrupting the continuity of nature and the ubiquity of physical law. Contrary to Canguilhem’s earlier biologically foundationalist hostility to it as well as to latter-day processual vitalism, ‘classical’ vitalism can lead to a kind of disciplinary pluralism or disciplinary division of labour rather than competition over the same disciplinary territory; and the physical sciences stand no more chance of holding the entire field any more than do the sciences of life. For instance, Schrodinger’s formal characterization of life – like the work of Canguilhem himself, dating from the 1940s and taking its inspiration from contemporary physics – as a kind of self-organizing negative entropy able to generate order from order – hardly disqualifies, for all its foundational qualities, the independence of the activities of biology (Schrodinger 1992). Disciplinary foundationalism and outright imperialism are not the same thing.

More recently, the category of emergence – the idea that an outcome can be more than the merely mechanistic sum of its constituent parts – has demonstrated the extent to which the actual existence of biological properties is not inexplicable on the basis of what we know about physical laws and mechanisms whilst still remaining a specific, disciplinary stance proper to biology itself (Johnson 2001; Weber 2010). But equally the category of emergence does not necessarily lead to conclusions of a processually vitalist kind. The existence of emergence in a variety of non-living systems or at least systems that are composites of the living and the physical worlds (such as cities) does not mean that all systems can – along the lines suggested by holistic vitalism – be reduced to abstract categories held in common, only that vitalism and mechanism are no longer necessarily mutually exclusive opposites. The very terms of the debate have quite obviously changed, but not to the detriment of the continuing relevance of Canguilhem’s kind of vitalism. Emergence is a normal aspect of natural processes, physical or vital. Life itself was no doubt originally the product of emergence. Biological systems theory has posited that life, under appropriate initial conditions (which may themselves be improbable), is itself probable rather than improbable (Maturana and Varela 1998). With the concept of emergence, it is now possible to comprehend how something like the unity of the cell or even the organism can emerge out of disparate processes and systems, without there being a need to posit a processually vitalist ghost in the machine. Generalising, processual vitalism is – in Howard Caygill’s terminology (referring to Whitehead) – an *analogical* form of discourse whereby matter is understood to be processual by analogy with life; analogical, to put it crudely, in that if both nature and the living are processual and vitalism is a form of thought that highlights the processual, then all of nature can be understood according to the principles of vitalism. But the fact that life and matter are related by analogy, via in this case the concepts of event and process, does not mean that they are the *same* or even parallel. Rather, they are at most *comparable*. Little could be less akin to Canguilhem’s own view of the originality of life, and the vitality of vitalism that stems from it, than Whitehead’s processual subsumption of life and non-life out of this analogical relation (cf. Caygill 2007: 19–20).

For Whitehead an organism’s ‘nature’ was inseparable from its environment; and just as for an organism there is the organism itself and its environment so for an electron there is the electron and its electromagnetic field. Thus the so-called ‘philosophy of organism’ was an attempt on the part of Whitehead to extend the notion of organism to all of nature, with the consequence that all ‘concrete enduring entities are organisms’ (Whitehead 1967: 79; cf. Whitehead 1978, esp. chapters 3 and 4; Stengers 2011: 166, 173–4). This is evidently a generalising, processual model rather than a disciplinary model: the fact that the biological organism was the ‘original’ model for the philosophy of organism more generally does not imply that life, for Whitehead, was ‘originating’ in Canguilhem’s sense; hence, indeed, Whitehead’s designation of his philosophy as a kind of hybrid ‘organic mechanism’ (Whitehead 1967: 80). As such, Whitehead was not really a vitalist at all; or at least if he was a vitalist it was in his sense that ‘nature is alive’ *in toto* with no particular privilege for the living being.

However, for a vitalist like Canguilhem this would not necessarily lead to the conclusion that such processual analogism as that of Whitehead is itself ‘false’. It is rather to suggest that the impulse to such varieties themselves may be something like an inevitable temptation or extrapolation from the ‘original’ originality of life. Life is, so to speak, symptomatically susceptible to generalizing and expansionist extrapolations of itself. Just as, for Canguilhem, technique is to be understood as ultimately a vital fact, so should the will to think of myriad phenomena in terms of the processual affectivity of becoming be considered an extrapolation from the originality of life itself. Nonetheless, that originality does, for him, imply a disciplinary privilege. Generalising processualism is a temptation that relies upon the model of the organism without according it, for all that, any originating privileges. All of nature in Whitehead is subject to the ‘evil’ effect of time, the terror of loss (Whitehead 1978: 340). But there is nothing *specific* to the living organism here. Biology and medicine have no disciplinary priority; his organisms are generalized relations or ‘societies’ not living beings in a particular kind of predicament – that of the susceptibility to ‘error’, sickness and pathology.

## The Organism and (Non-)finalism

Canguilhem’s vitalism was of course never an *in*organic one. We do not have the post-Deleuzian paraphernalia of the body-without-organs and such like, nor, as we have seen in Whitehead, the generalization of the concept of the organism itself to mean just about any entity in living or physical nature (Deleuze and Guattari 1989). Influenced so powerfully by Kurt Goldstein, the specific living organism was really the horizon of all of Canguilhem’s thought in this area (Canguilhem 1989: 181–96; Cf. Goldstein 1995). Life, then, for Canguilhem is organic ‘totality’; but in so far as it is totality it is also *polarity*. The organism is only such in relation to an environment with which it interacts, and (following von Uexküll, and Goldstein himself) a milieu which it establishes; and this environmental relation is dynamic, *polar* – a matter of struggle and of the permanent, threat of disequilibrium, breakdown and ultimately extinction. Hence the crucial importance of the notion of ‘milieu’ for Canguilhem (Canguilhem 2008a). As with von Uexküll, the milieu is, so to speak, ‘extracted’ from the environment; the *Umwelt* is an ‘elective abstraction’ from an environment, an *Umgebung* (Canguilhem 2008a: 111). It is difficult not to think here of the famous tick, described by von Uexküll, with its constricted milieu of the branch, the blood, the falling from the branch, laying of eggs, then death (von Uexküll 1957: 6–7; and see now the translation in von Uexküll 2010).

For Canguilhem, what is involved here in the idea of the *Umwelt* is what he terms *centration* on an environment, a sort of pulling of the environment by the organism into itself (Canguilhem 1989: 284; cf. Le Blanc 2002: 225–234). Or as Goldstein has it (1995: 85):

The environment of an organism is by no means something definite and static but is continuously forming commensurably with the development of the organism and its activity. One could say that the environment emerges from the world through the being or actualization of the organism.

In this sense, just as for biosemiotic research more generally, life – and Canguilhem’s famous (originally 1945) essay on cell theory is also very much pertinent here – is itself a *relational* rather than a substantive phenomenon (Canguilhem 2008a: 25–56; Kawade 2009: 206–207). Further, life from this sort of perspective is inherently perspectival, even in a sense ‘biased’. And here perhaps is the real originality of Canguilhem’s vitalism, what we call its pathic dimension. As living beings we have to take the side of life, against disease, entropy, death. We ‘choose’ life. This is why medical technique was of such *existential* import for Canguilhem (Canguilhem 2011). In terms of the human organism, the doctor is always on the side of life, aiming to restore the organism to relatively balanced norms even if these are less than fully ‘normative’. In this sense sickness is not an absence of ‘normal’ functioning but the advent of a more constricted normative world.

For the physician, life is not an ‘object’ to be preserved but a polarized activity, whose spontaneous effort of defence and struggle against all that is of negative value is extended by medicine by bringing to bear the relative but indispensable light of human science (Canguilhem 1989: 131).

The doctor does not ‘normalize’, but attempts to assist the substitution of a preferential (albeit generally diminished) vital order for a less preferential (diseased, defunct) one. The doctor sides with the o*rganizational* integrity of the human being, however limited and deficient; it is, as it were, the organization of the organism that is decisive, but not some humanistic conception of the whole person.

Organic ‘organization’ is however a vitalist notion here, even if it offers, so to speak, concessions to mechanism (cf. Bechtel 2007). The organism is organized to exist in and to organize a particular milieu, but this does not mean that the organism is ‘adapted’ to the milieu in a ‘finalist’ way. This, again, is what separates the organism from the machine; latitude, non-finality, and – because of the ‘non-adaptive’ gap opened up between means and ends – the possibility of error, of *pathos*. Machines at least as they currently exist tend to have finalised ends; they exist for some or other determinate purpose (Kawade 2009: 211). Living organisms do not have finality in this sense, unless one is to engage in the kind of tautological metaphysics about genes that has been common in popular science over recent decades. But if living organisms are machines for perpetuating genes, then what kind of finalism can we apply to genes themselves? Organisms may be purposive, as the biosemiotic tradition suggests, but for all that we simply do not know what living systems are *for*. Indeed, as recent biosemiotic concepts such as ‘semiotic freedom’ indicate, living beings are purposive but not finalized (Hoffmeyer 2011). In some very striking paragraphs, Canguilhem insisted that the idea of the finality of the organism acknowledged its own limitations precisely in expressing itself. This is an aspect of organic life not characteristic of the non-organic dimension, and also at some considerable remove from the more affirmative tendencies of generalizing, processual vitalism; it is to emphasise indirection, fragility, pathos.

For Canguilhem, life is that which is capable of error, a theme which Michel Foucault located at the very core of Canguilhem’s vitalism, indeed of his entire thinking (Foucault 1989: 22; Canguilhem 1989: 275–287). Living beings have a kind of ‘non-final’ finality. Instead of an ontological finality, Canguilhem defends the idea of an *operational* one. Life’s failures and pathologies do not refute such an operational finality but endorse it.

If there were a perfect, finished finality, a complete system of relations of organic agreement, the very concept of finality would have no meaning as a concept, as a plan and model for thinking about life, for the simple reason that there would be no grounds for thinking in the absence of all disparity between possible organization and real organization. The thought of finality expresses the limitation of life’s finality (Canguilhem 1989: 281).

The living being that is capable of error (‘l’erreur’) also signals the status of the living being as a kind of waverer or subject of wandering (‘l’errance’) without determinate, ultimate finality. Human beings are no doubt distinguished, in so far as they can be distinguished, by being the least finalised of all. They make mistakes partly because like all living beings they *are* mistakes – ‘normalized monsters’, as Canguilhem calls them – but also because they do not know necessarily where to put themselves (Canguilhem 1968a: 364).^10^ If animals compensate for the possibility of error with instinct, humans compensate for it by always seeking out further information, by attempting to define obstacles so as to overcome them, always to correct error. And this after all is what science *is* for Canguilhem as much as it is for Peirce, Popper or Lakatos: the critical correction of error on the basis of the constant encounter with obstacles and problem-solving. To be alive is to be dissatisfied, argues Canguilhem; it is not to know quite who, what or where one is. But of course some kinds of organism are fated to have more operational latitude than others; and especially with the human living being, there is, for Canguilhem, just as for latter-day biosemioticians such as Jesper Hoffmeyer and others, no ‘final’ finalism.^11^

## Error, Pathos and Sickness

Proneness to error is not least, for Canguilhem, what once more sets animal organisms but above all humans apart from machines (Canguilhem 2008a: 75–97). Machines break down but they are not subject to errors as such. Computers can compute but they cannot *think*. They do not ruminate vaguely and indeterminately and more or less *a propos of nothing* as Newton did before his great discoveries. ‘What is this situation of thinking at which one aims at what one does not see?’ (Canguilhem 2008b: 12) We have all experienced how invention, creativity and inspiration can transpire from thinking *laterally*, thinking about something else other than the immediate problem. ‘Invention’, says Canguilhem, ‘cannot exist without consciousness of a logical void, without being drawn to a new possibility, without the risk of being mistaken’ (2008b: 12). Machines can be broken but they cannot be mistaken in this sense, and they do not fall sick. In other words, there is no *pathos* to the machine; a machine cannot be ‘pathological’. A machine does not make its milieu, even if it has an environment – and a fairly recent study in fact appears sceptical that we might at present conceive of a robot with an *Umwelt* (Emmeche 2001).

For Canguilhem, at any rate, the existence of machines is dependent on – not just analogous with – the existence of organic life, and the study of technology is ultimately a sub-field of biology (Canguilhem 2008a: 96). Prioritisation or origination is not the same thing as analogy or alignment. The originality of life asserts its priority. Prioritization is founded on life’s basic originality rather than its inimitability; machines are imitations of organisms not instances of organic life. And if machines are ever developed that can be pathological, that can reproduce monster versions of themselves as well as clones, machines that can think and use (in this sense of ‘improvise with’) language, even machines that can reproduce, mutate and vary, machines that exhibit genuine powers of autopoeisis, this will be not just because it is living beings – ourselves – who have made them, but because life itself was original, i.e. the originating model for the making of such monsters, their inspiration. Such machines, whether considered to be alive or not by the jurists of bio-philosophy, would be suffused with principles derived – extended – from life as an original fact.^12^

The pathic principle – that life is subject to sub-normativity, to pathos, disease – is crucial. Canguilhem repeatedly stresses that only the biological organism is capable of falling sick, that pathology is always ‘existentially first’ and at the heart of any rigorous as opposed to any merely analogical, extrapolative or generalizing vitalism (Canguilhem 1989: *passim*; Canguilhem 2008a: 121–133). Machines do not succumb to pathology, nor are there in fact monster-machines. ‘Physical nature’ does not fall sick, unless specifically perceived in terms of the organism as in Lovelock’s morally worthy yet epistemologically dubious Gaia hypothesis. Canguilhem’s is a vitalism that is situated upon the organism specifically – whether that is the micro-organism, the animal organism or the human organism. But if organisms are generically fallible and subject to normative diminishment – if in this sense they are *pathic* – then organisms are likewise, and correlatively, *normative*; when healthy they have operational latitude, when sick or unhealthy they operate according to a reduced level of norms. Sickness is not absolute disorder; it is merely a reduced order, or rather a different – diminished – order. Moreover, it is not this or that part of the body that falls sick but the organism itself, given that the organism is a totality of organization not just because it is ‘self-organized’ but because it exists as a totality in relation to a milieu or an environment. Its individuality (a key term for Canguilhem) is, then, perspectival, relational (cf. Kawade 2009: 206–207). The organism as an agent of *centration* is focused upon a milieu that it ‘cuts out’ or extracts from its environment and which it attempts to hold stable against competition, transformation and disorder (Canguilhem 2008a: 111–112; von Uexküll 1957). Biosemiosis is obviously central to all this. Living beings signal to themselves, to each other and to the environment not as an aspect of what it is to be living but in ways that are constitutive of it. Life, observes Canguilhem, *radiates* (2008a: 114). But radiation is also, in a sense, domination, the will to power. By making up a milieu the organism imposes itself on the environment, it ‘ingests’ aspects of the environment, as it were, into itself. As we noted earlier, Von Uexküll distinguished between the *Umwelt* and the wider environment, the *Umgebung*, a distinction endorsed and adapted by Canguilhem; the environment is, so to speak, what is outside whilst the *Umwelt* is an external milieu (analogous to the internal milieu described by Bernard), but one on which the organism centres or ‘centrates’ itself.

For Canguilhem at least, organisms obviously live in tension as well as in alignment with their environment; all equilibrium in living systems is tentative and provisional.^13^ There exist critical conjunctures where the environment, as it were, overwhelms the milieu and the organism is stranded without a normative horizon. When the entire relation between the organism and the environment breaks down the organism enters into what Goldstein famously called the catastrophic situation, the response being a form of ‘catastrophic reaction’ or normative disarray (Goldstein 1995: 48–9). A human being overwhelmed by disorientating levels of stress and anxiety is, relatively speaking, within a catastrophic situation. Usually, some sense of order can be restored. Some catastrophic situations, however, are marked with ‘no exit’ signs. A laboratory animal isolated from its centrated – ‘natural’ – milieu and transplanted to the laboratory environment is in a catastrophic situation that is more or less irreversible (Canguilhem 2008a: 113). A laboratory mouse cast into a water tank in order to determine levels of ‘stress’ is not just in an experimental situation but in a catastrophic one; the animal has lost its milieu and is dominated by the extraneous situation of the laboratory.^14^ The laboratory is the milieu of the scientist not the animal. Here, we have the difference between a pathic situation which is always prone to error, which can always go wrong, which can always lead to a diminishment of agency and latitude and a catastrophic one where, so to speak, none of the usual rules apply; not merely a question of a lesser order but, from the perspective of the living being in question, a comprehensive *disorder*.

All this, perhaps obviously enough, takes us a long way from generalizing, processual kinds of vitalism. Processualism, here, is usually related to a celebratory kind of affirmation. In Deleuze’s work, for instance, we have a sort of cosmic, apparently celebratory affirmation undertaken in the name of Nietzsche: do not succumb to the *ressentiment* of the denial the world, instead affirm it! (Deleuze 2008). Indeed, Nietzsche’s vitalism – in contrast to that of Schopenhauer – is often held up as a vitalism of celebratory affirmation, but this is misleading. It depends what one means by ‘life’ and of course on what one means by affirmation. Georg Simmel observed that: ‘According to Nietzsche we will because we live, according to Schopenhauer we live because we will’ (Simmel 1991: 76). For Nietzsche willing is as much a *symptom* of life as a moral affirmation of it. What is affirmed when Nietzsche affirms life is life as *pathos*, as overcoming, struggle and experience. Hence a Nietzschean outlook is one that is capable of living up to *pathos*, of being equal to it such even that one *wills it*; hence the extraordinary but in this sense necessary doctrine of the Eternal Return, the limit-point, surely, of the very principle of Nietzschean *willing*.

In this Nietzschean sense – albeit one with a different emphasis from that of, say, most of the voluminous commentary on Deleuze if not perhaps of Deleuze himself (who was after all as influenced by Nietzsche as much as, if not even more than, Canguilhem) – we might say that life is affirmation in that it entails valuations in relation to an environment: the organism is committed to asserting itself in relation to an outside order or disorder. But such a commitment is more something to which we – as organisms, as humans – are condemned as *fate* than it is an affirmative choice, still less an affirmative, celebratory ‘choice’. Much here depends on how one understands the meaning of affirmation: whether as an ‘inflationary’ tendency that ‘celebrates’ the expansivity of life, or in a more value-neutral sense simply as the way in which life ‘expresses’ itself. Recall Canguilhem’s depiction of what vitalism *is*; an ‘exigence’, a duty or an obligation for the doctor or biologist at least, but not a substantive, positive *morality*. Who after all chose life? It is inflicted upon us; and few avowed vitalists could be further from the kinds of ecstatic, self-consciously affirmative vitalism that we have witnessed over recent decades in philosophy than Canguilhem.^15^ To put it baldly, one can be a vitalist and still think that life generally leaves much to be desired. For Canguilhem, vitalism was disciplinary not generalizing or processual; a matter of the organism, not some general transcendental, processual principle of non-organic, affirmative life. A vitalism such as his is inevitably pathic rather than celebratory or affirmative in the moral sense. Organisation and the organism retain their importance and we are never far from the recognition that vital organization can fail and that organisms fall sick and die.

This means that there is nothing intrinsic to vitalism to *justify* life in the affirmative, celebratory sense other than the original fact of life itself. If life is justifiable it is only in so far as life is original and originating, in other words it is only because justification itself *presupposes* life. ‘It is because value is in the living being that no judgement is made on it’ (Canguilhem 2008a: 125). In itself, life is as much a death sentence as a principle of cosmically vitalist, celebratory *joie*-*de*-*vivre*: if life, as Bichat thought, is all the resources that resist death, then we all know who or rather what is going to win out in the end. Life can fend off the consequences of the second law of thermodynamics but it cannot defeat those consequences. But then here again is the unavoidable, pathic originality of life itself. Life has absolutely no justification other than itself. It begins and ends only with itself. Vitalism is vital simply because it *has* vitality, and – however tautological it is, however ultimately bleak – that is all one can say.

However, this emphasis on these pathic aspects of life should not lead to an alternative morality of outright pessimism or miserabilism. It is not to replace an emphasis on the ecstasies of Nietzsche with the miseries of Schopenhauer but to replace, if anything, one kind of Nietzsche with another. Although a long way from affirmative, celebratory vitalism, Canguilhem himself clearly associated, in a broadly Nietzschean way, the originality of life with the values of creativity, open-ness, experiment and tolerance of variety.^16^ Non-finality, pathology, error – these have their upsides. Error produces change, variety and difference. Every form of ‘normal’ life was originally an error. And because life is subject to pathology and diminished normative order, the adventure of experimentation has to be an ethos that is derivative of life itself. Hence the doctor is inevitably an experimentalist in trying out this or that solution to the problems of sickness (Canguilhem 1968b: 389); and by extension life itself in constantly searching for solutions to problems of diminished normativity and entails experimentation, trial-and-error and a non-finalized openness to the future. From most forms of pathology, even from some catastrophic situations, it is possible to retrieve some new sense of vital order, some sense of normativity, albeit in many perhaps most cases more than likely a diminished one (Goldstein 1995: 49).

## Plasticity, Lability and the Brain

For another thing, there is organic plasticity; meaning is *open* when it comes to life. In biosemiotic terms, it is the constant striving of living beings to establish and maintain their varieties of *Umwelt* that requires, in even the simplest life forms, a degree of semiotic latitude to cope with different kinds of event. Machines do not have ‘latitude’ in this sense. The ‘meaning’ of a machine is whatever the machine is built to do, but the organism has latitude of meaning (cf. Canguilhem 2008a: 113; Goldstein 1995: 388). That is presumably why there is a biosemiotics on the one hand and communications ‘science’ on the other, but not a machinic-semiotics as such. Signs and signalling are phenomena of milieus not of finality and determination. Life experiments but also re-invents and re-interprets; it is marked by operational and cognitive plasticity, or what Canguilhem at times refers to as ‘lability’ (Le Blanc 2002: 119–20). The same organism can exist in a variety of milieus, even if it has a preferred milieu; and none more so than the human.

Yet Canguilhem’s vitalism is no humanism. Again, materialism and vitalism go together, and in a way which is closely connected to the pathic aspects of that vitalism and, as we shall see, to further aspects of the thought of Nietzsche. Above all, here it is the brain that is decisive in terms of lability or plasticity. The brain is sometimes treated in the philosophy of mind as if it itself were an organism (a sort of internal homunculus which itself thinks and acts), rather than as what it is, an organ within the organism, within the totality of the active, thinking living being (Read 2008). The neurosciences themselves invoke the brain as part of a wider, distributed system of interaction with an external milieu.^17^ In this sense ‘consciousness’ – still less ‘mind’ – is not restricted to the brain. It could be said to be the nervous system as a whole that ‘thinks’, that interacts with the environment and cognizes a milieu. We ‘think’ with or rather through our brains but also, and as a result, with our fingers, our stomachs, our eyes and our ears.

The brain is, obviously enough, a biosemiotic organ (Cariani 2001). It is the variegated and organising centration principle of the nervous system; in a sense we are ‘thinking’, just as animals are thinking, when the brain signals movements and reactions to stimuli in the environment (which is, after all, easily the bulk of what the brain does), just as we are thinking when we ponder the forces of gravity or whether to make a decision to dine at home or at a restaurant. We do not need to invoke the humanisms of consciousness here, only the variegation of life itself. Conscious thought is not something exceptional, transcendental or even uniquely vital – still less uniquely human – but is a variety of biological information or signalling, including self-signalling to the ‘internal milieu’ of the brain itself, albeit a variety that is both highly specialized (evolved), open-ended and ‘facultative’.

If the brain is a certain kind of vital sensor, its components have been added on, as it were, here and there at different periods and according to differing demands. Were it a machine at all it would be a Tinguely machine not a metronome or a clock. The philosopher Bernard Williams captured this idea in his conception of the human organism as a whole as something of a *mess*, with different strata added on at different times and not necessarily adding up to something uniformly un-contradictory or ‘adaptive’ (Williams 1995: 108). But this is especially so of the human brain. The brain is, as it were, *more* of a mess than the rest of the human organism, with the consequence that there is no one-dimensional normality to the brain as a whole. One cannot straightforwardly read off ‘correct’, finalized norms from the brain because the brain throws up myriad and often mutually contradictory norms; norms of consumption for example (what makes the brain happy? – love, drugs, alcohol, all of which can definitely be ‘bad’ for the brain) (Schelling 1988); norms of regulation (digestion, secretion, homeostasis); norms of habit (synaptic repetition that can lead to dead ends – addiction – but also to intelligence and innovation, sometimes *via* dead ends like addiction); norms of self-preservation (warnings, flight impulses) and so on. The brain is alive but that does not mean that it is uniformly adaptive or uniformly ‘rational’ in any easily-aligned sense.

As a variegated, distributed, ‘disrupted’ organ the brain is inherently pathic. Aspects of it – aspects of *us* – suffer or are diminished in relation to other aspects. We are condemned to an excess of consciousness, claimed Nietzsche. As an organ the evolution of the brain has enhanced, as it were, the scope for error of which the organism as a whole is capable; the evolved human brain makes humans, so to speak, *more capable of error* than ‘lower’ forms of life and so also capable of a wider variety of options, possibilities and ‘ways out’. Here vitality is, as ever, but more than ever, closely analogous to what is known often rather misleadingly as creativity. And creativity is aligned with a certain level of redundancy; both in the sense that there is a great deal of material in the brain that is probably not obviously doing very much in an immediate sense, and in the sense that much of what the brain does is idling, *not* thinking, or at least thinking in a highly latent state, but one which can ultimately be highly creative – as in Canguilhem’s own invocation of the example of Newton:

When Newton was asked how he found what he was looking for, he is alleged to have responded: “By thinking on it continually”. What sense are we to give to this “on”? (Canguilhem 2008b: 12; cf. Canguilhem 1996).

There is no such thing as a creative machine. The brain is not divided between the vital and the mechanistic; rather its mechanics are if anything hyper-vital; or, expressed in a more deflationary way, the gap between functionality and finalism is wider here perhaps than anywhere else. To those who claim that consciousness is not a problem, the vitalist response would be that the brain itself is a problematizing organ (cf. Read 2008). Consciousness is a fact of life, which is to say – not just functional regulation, not just self-awareness, but the invention of norms, sometimes imaginary norms, projected onto the environment, the confrontation of obstacles, projection on to a milieu, and constant potential for *error*.

## Nietzsche, Vitalism and Power

The possibility of error, then, is connected to vital ‘affirmation’ itself, that is, to what Nietzsche called power. Again, the Nietzsche we would want to invoke is not the celebratory, delirious one favoured by some varieties of post-Deleuzian cultural theory but, as it were, a more deflationary Nietzsche. For Nietzsche *pathos* is opposed to *ethos*, where *ethos* denotes the continuity of life, whether as being or becoming, and *pathos* denotes the occasions and challenges through which any life passes through (see Strong 1975: 233–4; cf. Le Blanc 2002: 355–8). *Pathos* is related to what happens to us, to error, to pathology, but it is also, as Tracy Strong shows, closely linked to the will to power itself since the will to power is what aims to overcome or at least live up to *pathos*. Understood as the attempt to incorporate all that we encounter into form, the will to power might be described actually as the sum of our *pathos*, our individuality: ‘All the forms a thing acquires constitute its *pathos*, its will to power’ (Strong 1975: 234). Life, for Nietzsche is, in Strong’s words (1975: 233 & 235):

the attempt to give one’s characteristic definition to that which is encountered… A living organism tends then to attempt to organize the world around it in its own image and to make it part of the sphere of its domination.

In the text known in English as *The Gay Science*, Nietzsche invoked *pathos* as a past experience, a passion that cannot endure perpetually but which can be invoked in recollection (Nietzsche 1974: 252). *Pathos* and experience are closely linked; just as for Canguilhem ‘le vivant’, the living, is closely related to ‘le vécu’, the *lived*. This reminds us that, perhaps contrary to the forms of post-Deleuzian vitalism that we invoked at the beginning of this paper, there is no abstract ‘life’, no generalizing, processual *élan vital*, no vitalism without localization and organization, no vitalism without the organism; and so no *generalized* processual, vitalist becoming but only the particular and accumulated *pathos* of life in its many aspects plus the motley processual cosmos of non-living forces and entities. Integral to the originality of vitalism is that vitality itself should not be a general phenomenon but, in each form, an *original* fact. That is not least what we have meant in invoking a disciplinary rather than a generalizing or processual vitalism. This vitalism, if it is itself to have *semiosis*, i.e. semiotic and critical leverage – or in other words *meaning* – registers the particularity and originality of life specifically, not the general processual-becoming of all things living or non-living.

Vitalism, then, is nothing if it is not discriminating. In this spirit, Nikolas Rose has proposed the idea of a ‘vitalism test’ to gauge the relevance of experimental activities in the laboratory.^18^ Two closely-related aspects of this are of particular importance; that matter is alive when it is organized by and organizing its milieu, and that matter is alive when its forces are organised to resist death. One might envisage a parallel sort of vitalism test applied to types of thinking in the social and human sciences. Do such vitalisms apply to forms of matter and organization that are specifically organized against death and which engage in a polarized relation to a milieu that is both determining and determined? If not, one is hardly respecting the originality of life, only extrapolating from it in an easily processual, inflationary, one-dimensionally – celebratory – affirmative way. Life itself is not the celebration of process; it is an accident, usually a mess, it is error, inadequacy, provisional equilibrium, technical improvisation, and cognitive experimentation involving what is necessarily always a half-blind, perspectival and fallible groping towards operable facts and concepts; facts and concepts which turn perpetually inwards and outwards upon themselves as life informs and is informed by the fragile and tentative conditions of its own originality.

## Conclusion

The aim of this paper has been to assert – especially via the writings of the historical epistemologist and philosopher, Georges Canguilhem, and in opposition to more generalized, processual notions of life as becoming – the specificity of the living being as the basis of any renewed vitalism today. The paper also argued against the more ‘celebratory’ aspects of generalized, processual kinds of vitalism in positing a view of life as *pathos*, as differentiated ‘affirmation’ and potential for pathology and sickness.

## Footnotes

1 This passage – taken from Nietzsche (2003: 247) – is helpfully discussed in Strong (1975: 233–234), and also in the final section of the present paper.

2 Although Magnus (2008) restricts his use of the term to the particular period 1920–1940, his ‘holistic-disciplinary’ distinction has been an important influence in the structuring of the argument of the present paper. Even more important have been the comments of Nikolas Rose and, above all, the very detailed and challenging comments on an earlier draft from two anonymous referees from this journal. I have tried as best I can to meet all of their objections and to follow their suggestions in the final version.

3 Greco’s account, in a largely processually-oriented collection, is actually ‘disciplinary’ in emphasis, as is in keeping with its focus on Canguilhem. For a still very serviceable account of Canguilhem’s thought, see Gordon (1980). I have also found very useful two historically and philosophically well-informed papers by Charles Wolfe (2015, Wolfe and Wong 2014). See as well the cogent and informative introduction to the English-language translation of Canguilhem’s *Knowledge of life* (2008a), by the translators.

4 Richard Fortey’s book *Life*: *an unauthorized biography* (1998) manages to give a comprehensive descriptive account of terrestrial life without troubling too much about definitions.

5 In *La connaissance de la vie*, Canguilhem invokes Emmanuel Rádl in referring to vitalism as a ‘morality’ – *morale* (Canguilhem 1965: 88) – a term which is translated not entirely inaccurately in *A vital rationalist* (1994: 288), as ‘ethical system’. Canguilhem himself describes vitalism as an ‘exigence permanente de la vie dans le vivant’. This might be glossed (rather than translated) as the ‘persistent obligation that life demands of the living being.’ In any case, *exigence* is not just *ethos* or morality, but suggests obligation, demand, necessity…

6 The question of the influence of Bergson on Canguilhem still awaits an extended study. For now see Le Blanc (2002: 215–224), especially over the issue of creativity, and more generally Wolfe and Wong (2014).

7 These are of course themes that are to be found in different ways in the work of philosophers both prior to Canguilhem – Bergson – and more or less contemporary to him – Ruyer, Simondon. However, though influenced by all three, Canguilhem, in a style that is rather distinct from them tends to employ the register of the ‘infra-rationality’ of the biological and medical sciences rather than the establishment of a priori philosophical positions to arrive at these emphases.

8 Although as Monica Greco has shown, this stance is at least comprehensible within the terms of Canguilhem’s prioritisation of the living organism over the powers of mechanism (Greco 2005: 19–20).

9 Canguilhem does in fact invoke J.S. Haldane on three occasions in *The Knowledge of Life*, without nonetheless assessing his biological philosophy as a whole.

10 ‘Successes are delayed failures; failures are aborted successes… All living forms are, to use Louis Roule’s expression, in *Les Poissons*, “normalized monsters”. Or as Gabriel Tarde puts it in *L*’*Opposition Universelle*, “the normal is the degree zero of monstrosity”, with zero here meaning the vanishing point’ (Canguilhem 2008a: 126).

11 For a comprehensive discussion of the ‘anthropological’ dimensions of Canguilhem’s thought, see Le Blanc (2002). From Le Blanc’s book one gains the impression that just as Darwin’s problem in *The Origin* was very often to do with ‘man’ without mentioning him, so is Canguilhem’s own vitalist thought overwhelmingly preoccupied with human life.

12 Ian Hacking’s essay devoted ostensibly to the somewhat arcane topic of Canguilhem’s relation to the philosophy of cyborgs is more profitably read for its fundamental reflections on the idea of organic prosthesis, or extensions from the body (Hacking 1998: especially 204–206).

13 I am grateful to a referee from this journal for pointing out that in fact for von Uexküll the *Umwelten* are specific and, in that sense, conceived as un-changing rather than, as in Canguilhem’s own emphasis, more provisional or at least tentative.

14 I owe this reference of the laboratory animal in a catastrophic situation to Nikolas Rose. It might of course be possible to argue that the laboratory mouse is indeed precisely within its own milieu, given that it has been specifically bred for it and knows nothing else; the mouse in the laboratory is not exactly a mouse but something more like a mouse-clone. Existing in its own centrated milieu cut out from the natural environment the mouse is characterised by a host of individual differences, from one to another; yet laboratory mice are just about all the same, the most part having been bred from a single ancestor (Jones 1999: 64). This, one might suggest, makes the laboratory mouse actually attuned to the laboratory, but non-attuned specifically to what is supposedly being measured; the ‘wild’ mouse out in its ‘natural’ milieu. Linking of one mammal species to another (say mice to humans) is problematic enough, even without laboratory mice being so different from ‘normal’, normative mice. On the other hand, we would still want to insist, with Rose himself, that the mouse is in a catastrophic situation in the laboratory even if it knows no other situation; first because the truly catastrophic situation is the experiment itself rather than just life in the laboratory, second because phylogenetically the mouse, even if a clone was cloned from a mouse that was used to a very different kind of non-laboratory milieu. On issues of vitalism and animal experimentation (and Canguilhem), see the account in Rose and Abi-Rached’s book *Neuro* (2013: chapter 3).

15 On the other hand, there might be a case for claiming that even Deleuzian vitalism is based on a sort of pathos at one remove; one might speculate that because the organism falls sick and dies, Deleuze compensates by insisting on precisely a *non*-*organic* – which is equally a non-organismic – concept of life, one which is beyond the mere organism and is even against it. In any case, is it absurd to suggest that there is something depressing about endless Deleuzian celebratory affirmation, just as there is something life-affirming, heartily sardonic and jocular about the miserabilism of a Samuel Johnson, Schopenhauer, Dorothy Parker or Samuel Beckett?

16 Which is no doubt why some of the more interesting studies in the field of generalized, processual vitalism often relate precisely to the domain of creativity. See for instance the work of Mariam Fraser (e.g. 2005, and especially in the context of what we are calling pathic vitalism, Fraser 1998).

17 See for example Squire et al. (2008) for a standard rendition, demonstrating that neuroscience itself is not ‘cerebro-centric’ but concerned with the nervous system as a whole. Here ‘mind’ as a ‘problem’ simply does not appear.

18 Nikolas Rose, personal communication.
